# RETRACTED: Molagoda et al. GSK-3β-Targeting Fisetin Promotes Melanogenesis in B16F10 Melanoma Cells and Zebrafish Larvae through β-Catenin Activation. *Int. J. Mol. Sci.* 2020, *21*, 312

**DOI:** 10.3390/ijms262411832

**Published:** 2025-12-08

**Authors:** Ilandarage Menu Neelaka Molagoda, Wisurumuni Arachchilage Hasitha Maduranga Karunarathne, Sang Rul Park, Yung Hyun Choi, Eui Kyun Park, Cheng-Yun Jin, Haiyang Yu, Wol Soon Jo, Kyoung Tae Lee, Gi-Young Kim

**Affiliations:** 1 Department of Marine Life Sciences, Jeju National University, Jeju 63243, Republic of Korea; neelakagm2012@gmail.com (I.M.N.M.); hasikarunarathne@gmail.com (W.A.H.M.K.); srpark@jejunu.ac.kr (S.R.P.); 2 Department of Biochemistry, College of Oriental Medicine, Dong-Eui University, Busan 47227, Republic of Korea; choiyh@deu.ac.kr; 3 Department of Oral Pathology and Regenerative Medicine, School of Dentistry, Institute for Hard Tissue and Biotooth Regeneration, Kyungpook National University, Daegu 41940, Republic of Korea; epark@knu.ac.kr; 4 School of Pharmaceutical Science, Institute of Drug Discovery and Development, Zhengzhou University, Zhengzhou 450001, China; cyjin@zzu.edu.cn; 5 Institute of Traditional Chinese Medicine, Tianjin University of Traditional Chinese Medicine, Tianjin 300193, China; yuhaiyang19830116@hotmail.com; 6 Department of Research Center, Dong Nam Institute of Radiological and Medical Sciences, Busan 619953, Republic of Korea; sailorjo@dirams.re.kr; 7 Forest Biomaterials Research Center, National Institute of Forest Science, Jinju 52817, Republic of Korea; leekt99@korea.kr

The journal retracts the article, “GSK-3β-Targeting Fisetin Promotes Melanogenesis in B16F10 Melanoma Cells and Zebrafish Larvae through β-Catenin Activation” [1], cited above.

Following publication, concerns were brought to the attention of the Editorial Office regarding potential irregularities found in images presented in this article [1].

Adhering to our standard procedure, an investigation was conducted by the Editorial Office and Editorial Board, which confirmed multiple panel overlaps contained within Figure 2, presenting different experimental conditions. While the authors collaborated within this process, supporting material and explanations provided did not meet Editorial Board expectations in order to resolve the issues raised. As a result, the Editorial Board has lost confidence in the reliability of the findings and decided to retract this publication [1], as per MDPI’s retraction policy (https://www.mdpi.com/ethics#_bookmark30).

This retraction was approved by the Editor-in-Chief of *IJMS* journal.

The authors did not provide a comment on this decision.
